# CD44^+^/CD24^-^ Expression as predictors of ovarian cancer chemoresistance: immunohistochemistry and flow cytometry study

**DOI:** 10.1186/s43046-022-00143-2

**Published:** 2022-10-24

**Authors:** Unedo Hence Markus Sihombing, Andrijono Andrijono, Gatot Purwoto, Supriadi Gandamihardja, Alida R. Harahap, Primariadewi Rustamadji, Aria Kekalih, Retno Widyawati, Dzicky Rifqi Fuady

**Affiliations:** 1 Gynecologic-Oncology Division, Department of Obstetrics and Gynecology, Tarakan Hospital, Jakarta, Indonesia; 2 Doctoral Program in Medical Sciences Faculty of Medicine, Universitas Indonesia, Jakarta, Indonesia; 3 Gynecologic-Oncology Division, Department of Obstetrics and Gynecology, Cipto Mangunkusumo Hospital, Jakarta, Indonesia; 4 Gynecologic-Oncology Division, Department of Obstetrics and Gynecology, Hasan Sadikin Hospital, Universitas Padjadjaran, Bandung, Indonesia; 5 Department of Clinical Pathology, Faculty of Medicine, Universitas Indonesia — Cipto Mangunkusumo Hospital, Jakarta, Indonesia; 6 Eijkman Institute for Molecular Biology, Jakarta, Indonesia; 7 Department of Anatomic Pathology, Faculty of Medicine Universitas Indonesia — Cipto Mangunkusumo Hospital, Jakarta, Indonesia; 8 Occupational Medicine Division, Community Medicine Department, Faculty of Medicine Universitas Indonesia, Jakarta, Indonesia; 9 Department of Anatomic Pathology, Budhi Asih Hospital, Jakarta, Indonesia; 10 Department of Obstetrics and Gynecology, Karawang General Public Hospital, Karawang, Indonesia

**Keywords:** Ovarian Cancer, CD44^+^/CD24^-^, Flow cytometry, Immunohistochemistry, Chemoresistance

## Abstract

**Background:**

The conventional standard treatment for ovarian cancer is not very effective, and the disease is fatal for women. Cancer Stem Cells (CSCs) that express CD44^+^/CD24^-^ can contribute to chemoresistance and a poor prognosis. We seek to investigate the expression of CSCs (CD44^+^/CD24^-^) in ovarian cancer and their predictive significance.

**Methods:**

The ambispective cohort was performed on 64 patients (32 patients in each group) at four hospitals (Cipto Mangunkusumo, Tarakan, Fatmawati, and Dharmais Hospital). Debulking surgery was performed on the patients, followed by histopathological analysis. The patients had six rounds of chemotherapy and were under monitoring for six months. The therapeutic responses were evaluated using the RECIST criteria (Response Criteria in Solid Tumors) and categorized as chemoresistant or chemosensitive. Using immunohistochemistry, we directly assess the CSCs from ovarian cancer tissue and using flow cytometry to assess the CSCs from the blood.

**Results:**

High CSCs expression and ovarian cancer chemoresistance were significantly related in both trials (p 0.05). A better outcome was obtained using CD44^+^/CD24^-^ immunohistochemistry.

**Conclusions:**

We conclude that there is a substantial association between high CSCs expression and chemoresistance in ovarian cancer and that CSCs immunohistochemistry has a higher predictive value.

## Introduction

Ovarian cancer is a deadly women cancer [1]. Only 20% of women survive more than five years after being diagnosed with ovarian cancer, which has a risk of 1.3% [2]. Patients with ovarian cancer experience different symptoms that cannot be showcased to generate a definitive diagnosis. Therefore, further examination is necessary to identify ovarian cancer if symptoms such as abdominal distress or distension are detected [3]. Initially, 65–80% of patients respond to treatment, but 50% continue to develop platinum chemoresistance, which has a bad prognosis [4]. With standard therapies, twelve months of progression-free survival (PFS) and thirty months of overall survival (OS) would be achieved, which include debulking surgery followed by chemotherapy [5]. Chemotherapy resistance and a poor prognosis were caused by the cancer stem cells (CSCs) proteins. CSCs may be a target for cancer genetic therapy, according to recent studies [6].

The growth, metastasis, recurrence, and medication resistance of tumours are significantly influenced by cancer stem cells. Tumour recurrence was associated with high CD44 expression in chemoresistant ovarian cancer cell lines [6]. According to recent research by Klemba *et al.*, ovarian cancer with high CD44^+^/CD24^-^ has a stronger capacity for invasion and differentiation [2]. High CD44 expression was linked to chemoresistance, poor differentiation, high resistance, and recurrence rate, according to Zhang *et al.* [7]. Therefore, miR-199a's downregulation of CD44 in ovarian cancer may inhibit the cell cycle, diminish the ability for proliferation, and decrease invasiveness *in vitro* and tumour development *in vivo* [8].

The G1, S, G2, and M cell cycle stages are typical. Because the cells are not a part of the cell cycle but are still metabolically active, G0 is an early G1 phase and is referred to as the resting phase. The G1, S, and G2 phases are the interphases of cells, whereas the M phase is a mitotic phase (prophase, prometaphase, metaphase, anaphase, telophase). The G1 phase lasts the longest in normal cells, but CSCs have a shorter G1 phase that promotes rapid cell division to produce more stem cells [9]. The majority of chemotherapeutic targets are cells that are actively proliferating in the S or M phase [6]. CSCs, however, will stay in the G0 phase. CSCs' impact on the cell cycle results in chemoresistance of the cancer cells.

Identifying the association between CSCs and ovarian cancer chemotherapy response as well as the prediction capacity of CSCs is the goal of this project. We hypothesize that the CD44^+^/CD24^-^ has a connection to ovarian cancer and is a reliable indicator of chemoresistance.

## Methods

### Research Design

This is a two-year ambispective cohort study conducted at the Cipto Mangunkusumo Hospital, Tarakan Hospital, Fatmawati Hospital, and Dharmais Hospital (February 2018 - February 2022).

### Subjects

Patients with epithelial ovarian cancer in stages II-IV who agreed to participate in the study were the study's subjects. Pregnancy and other extra cancers met the exclusion criteria. Sixty-four patients made up the sample (32 in each group) using the consecutive sampling method. Thirty-two (50%) of the patients responded to the therapy with chemoresistance and 32 (50%) with chemosensitivity. For the flow cytometry and immunohistochemistry studies, samples were taken from every patient. After six months of surveillance, we observed no lost follow-up patients. Table 1 shows the distribution of patient profiles and clinical features.

**Table 1:** Basic patients’ characteristics

Variable	n (%)
• Chemoresistant	32 (50)
• Chemosensitive	32 (50)
Age (years old)	
• < 40	4 (6.3)
• 40-50	19 (29.7)
• > 50	41 (64.1)
Ca-125	
• ≤ 35	30 (46.9)
• > 35	34 (53.1)
Ovarian cancer stage	
• Early stage: II	5 (7.8)
• Advance stage: III - IV	59 (92.2)
Operation type:	
• Optimal Debulking	56 (87.5)
• Suboptimal Debulking	8 (12.5)
Differentiation/cancer grade	
• Good	13 (20.3)
• Intermediate	16 (25.0)
• Poor	35 (53.1)
Tumor histology type	
• Low-grade Serous	24 (37.5)
• High-grade serous	14 (21.9)
• Mucinous	3 (4.7)
• Endometrioid	12 (18.8)
• *Clear cell*	10 (15.6)
• Others	1 (1.6)
Lymph nodes metastasis	
• Positive	32 (50)
• Negative	32 (50)
Ascites	
• Positive	36 (56.3)
• Negative	28 (43.7)
Tumor size	
• 5 cm	17 (26.6)
• 5 -10 cm	15 (23.4)
• > 10 cm	32 (50)
Tumor residue	
• < 1cm	56 (87.5)
• > 1cm	8 (12.5)

### Data collection

The patients received histopathological examination and surgery. Patients who had malignant outcomes got six chemotherapy sessions followed by six months of monitoring. The effectiveness of the medication was assessed using the RECIST Criteria, after which it was divided into chemoresistant and chemosensitive responses. We performed retrospective immunohistochemical analysis of the ovarian cancer tissue and prospective flow cytometry to analyze CD44^+^/CD24^-^ blood expression. Serum Ca-125 levels, operation type, cancer histology type, cancer stage, tumour cell differentiation, cancer residue, cancer size, ascites, and lymph node metastases are among the other factors that were taken into account. The FIGO criteria were used to establish the disease stage. The Research Ethics Committee of Universitas Indonesia granted us ethical permission.

### Slide preparations

Eight 3 μm paraffin-block preparations were employed in this study to analyze the tissue. The blocks were then heated to 600°C and dried (30 min). We rehydrated it with serial alcohol, deparaffinized it using graduated xylol, and then washed it with water (5 minutes). Using 1.5 per cent hydrogen peroxide in methanol for 10 minutes, we isolated the area before washing it with water (5 min). To stop endogenous peroxidase activity, we applied a blocking technique. In the pre-treatment, a 960°C Decloaking Chamber contained Tris EDTA acid (10 min). We cleaned it in phosphate-buffered saline after cooling the blocks for 45 min (PBS). With the help of the Universal Background Sniper formula, we repeated the blocking procedure (15 min).

Using antibodies directed specifically against CD44 and CD24, we were able to identify their expression. We used a 1:500 dilution of the antibodies to incubate the preparations. After an hour, we washed it with PBS (5 min). Cocktail staining (double staining), was employed. After that, it was washed with PBS and incubated for 20 minutes with a Trekkie Universal Link solution (5 min). After that, we washed it in PBS and incubated it for 15 min with track-Avidin-HRP labelled (5 min). Diaminobenzene (DAB) was added to the 1 mL of a substrate, and it was vortexed (15 s). We drizzled it with the preparation, incubated it for two minutes, and then washed it (10 min).

After counterstaining with Counterstain Kit (CAT) hematoxylin for five seconds and washing with water, the preparations and substrate were examined (5 s). The preparations were submerged in distilled water at 5 % for 5 s before being rinsed with water (5 min). For dehydration procedures, we employed graded alcohol, and for cleaning procedures, graded xylol. A mounting solution and cover glass were used to finish the preparation. We decided to include stromal tissue internal positive controls and negative controls devoid of primary antibodies in each smear. To evaluate the preparations, we utilized a Leica ICC 50 HD microscope.

### CSCs immunohistochemistry cell counts

The CSCs' (CD44^+^/CD24^-^) expression was found by using a 400× light microscope magnification and three different fields of view. While the CD24 marker was red in both the cytoplasm and cell membrane, the CD44 marker was mostly brown in the cell membrane [10]. There is no red staining and a predominance of brown staining in the high CD44^+^/CD24^-^ cells. Low CD44^+^/CD24^-^ expression was defined as being less than 10% of the total coloured cells, whereas high CD44^+^/CD24^-^ expression was defined as being greater than 10% of the total colored cells.

### Flow cytometry methods

Peripheral blood samples as large as 5 mL were centrifuged, and the supernatant was then removed, leaving 50 μL of the sample to be resuspended. Fluorescent CD44 and CD24 antibodies were used to react with the samples (labelled PerCP and APC respectively). We eliminated the leukocytes from the pacific blue-labelled CD45 reagents. Then, we added 2.5 µL of CD44 and 2.5 µL of CD24 markers. The samples were first incubated for 15 min, followed by the addition of 300 μL of lysing solution and incubation. We repeated the centrifugation after adding 1 mL of FACS flow solution. After adding 500 μL of perm wash buffer and centrifuging the samples, we discarded the supernatant. We centrifuged the mixture after adding 1mL of perm wash buffer once more. Then, we added 200 μL paraformaldehyde (1% solution) in phosphate-buffered saline (PBS). We analyzed the samples with a flow cytometer.

### Statistical analysis

Data that were bivariate and univariate were examined. Chi-square or an alternate Fisher test were used to analyze the categorical variables. Both studies then investigated the AUC (area under curves) values and ROC (receiver operating characteristic) curves as a predictor of therapeutic response. In all research, we compared the power of CSCs using logistic regression.

## Results

### Ovarian cancer blood flow cytometry test

**Fig. 1:**
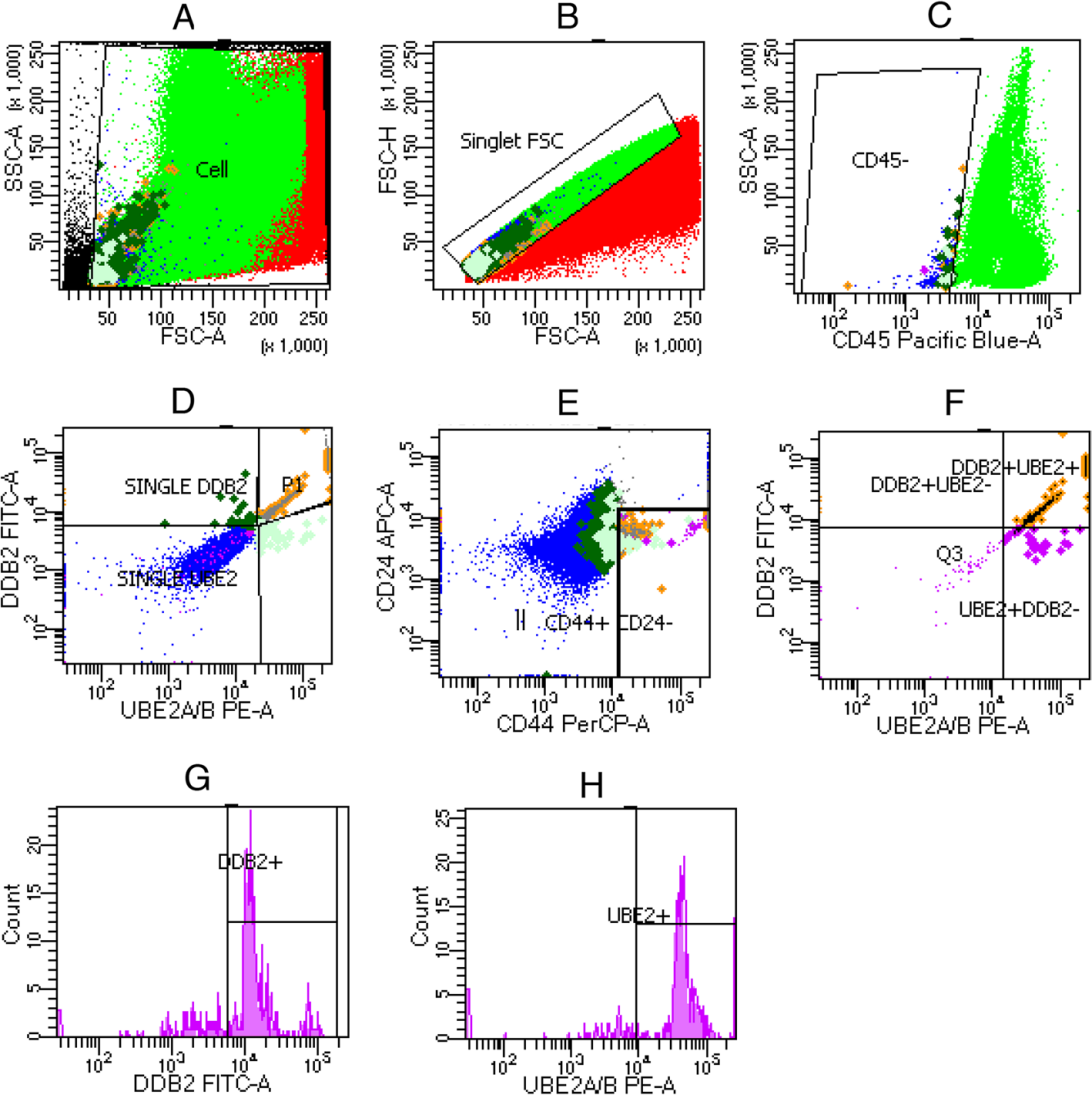
The flow cytometry pictures with automated flow cytometer. **A** Expression of total cells. **B** Expression of singlet FSC. **C** Expression of CD45. **D** Expression of UBE2A/B. **E** Expression of CD44 expression. **F** Expression of UBE2A/B. **G** Graphics of DDB2 cell count. **H** Graphics of UBE2A/B-cell count

**Fig. 2:**
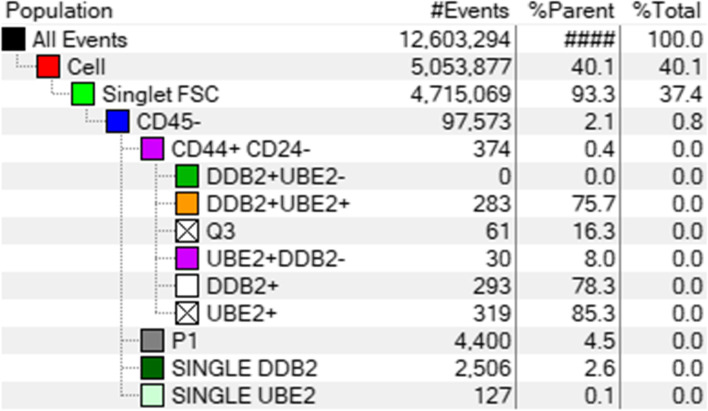
The flow cytometry cell count results. CD44^+^/CD24^-^ was calculated based on the proportion of purple cells from the Total Cells

By using a flow cytometer to measure the number of cells, CSCs were identified as those with positive CD44^+^/CD24^-^ marker expression. The percentage of the total cell count was used to compute the CSCs proportion. Figures 1 and 2 describe the flow cytometry analysis. The findings show that CSCs were substantially expressed in chemoresistance-positive ovarian cancer patients.

### CD44^+^/CD24^-^ Staining at ovarian cancer tissue

**Fig. 3:**
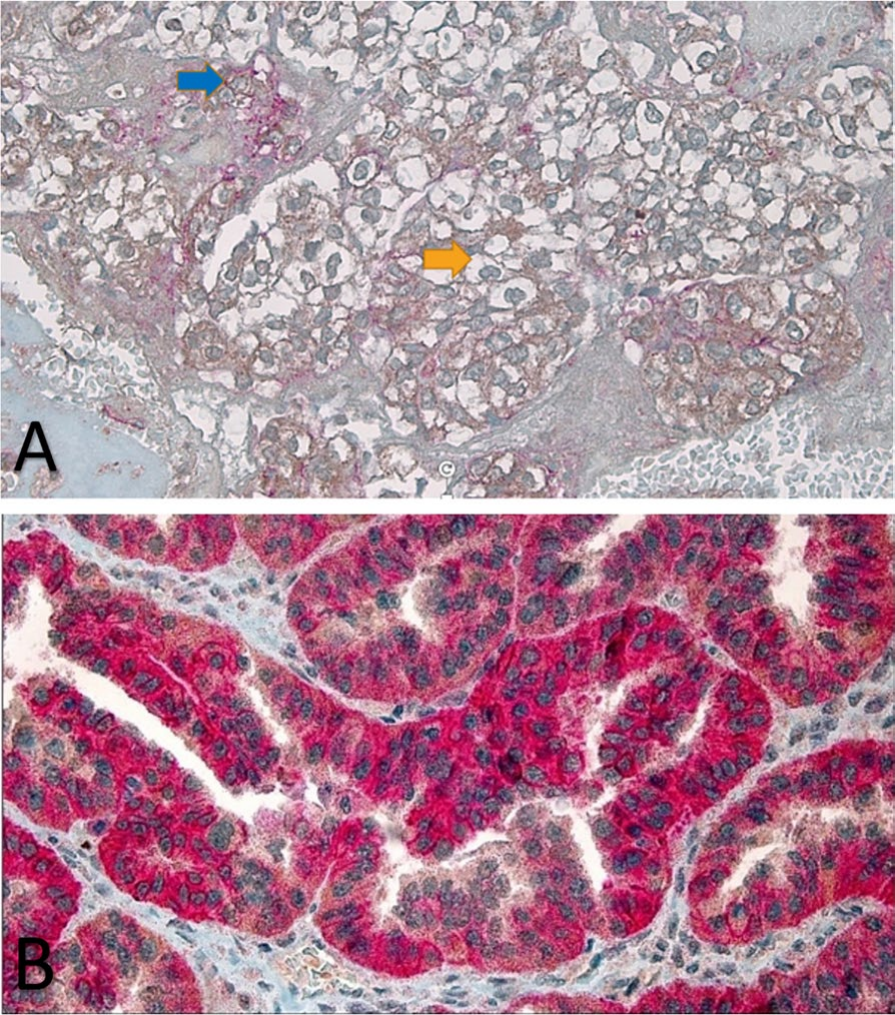
CD44^+^/CD24^−^ expression in ovarian cancer tissue (400× magnification). CD44^+^ cells have brown cell membrane (orange arrow). CD24^+^ cells have red cytoplasm and cell membrane (blue arrow). CD44^+^/CD24^−^ cells have a more dominant brown color than red color. **A** Chemoresistant ovarian cancer tissue, brown staining predominating over red. **B** Chemosensitive ovarian cancer tissue, almost all cells are colored red

When compared to CD24^+^ cells, which have a red cytoplasm and membrane (blue arrows in Fig. 3(A)), CD44^+^ cells have a brown cell membrane (400x magnification). In chemoresistant ovarian cancer tissue, there is significant CD44^+^/CD24^-^ cell expression, with brown staining predominating over red. Low CD44^+^/CD24^-^ cell expression is seen in chemosensitive ovarian cancer tissue in Fig. 3(B). It appears like most of the cells are red.

### Bivariate analysis

Some variables had results in Table 2 that were significantly different (p 0.05). Compared to the flow cytometry CSCs, the immunohistochemistry CSCs have an OR of 10.7 and 3.19. Immunohistochemistry CSCs hence have a higher OR and RR value. But CSCs immunohistochemistry and flow cytometry were reliable indicators of ovarian cancer chemoresistance.

**Table 2:** Research variables comparison based on therapy response

Variables	Therapy Response		*P* value	OR (95% CI)	RR (95% CI)
	Chemoresistant n (%)	Chemosensitive n (%)			
CD44^+^/CD24^-^ immunohistochemistry					
• High (≥ 10%)	26 (81.3)	3 (9.4)	0.001*	41.9 (9.5-184)	8.01 (2.7-23.6)
• Low (< 10%)	6 (18.8)	29 (90.6)			
CD44^+^/CD24^-^ flowcytometry					
• High (≥ 32692)	25 (78.1)	8 (25)	0.001*	10.7 (3.3-34.1)	3.19 (1.7-6)
• Low (< 32692)	7 (21.9)	24 (75)			
Ca-125 Level					
• ≤ 35	2 (6.25)	28 (87.5)	0.001*	105 (17.8-618)	7.93 (3.14-20)
• > 35	30 (93.75)	4 (12.5)			
Ovarian cancer stage					
• Early stage: II	1 (3.13)	4 (12.5)	0.162	4.42 (0.5-42)	1.68 (1-2.8)
• Advance stage: III - IV	31 (96.87)	28 (87.5)			
Surgery type					
• Optimal Debulking	25 (84.4)	31 (96.87)	0.023*	8.6 (1-75.3)	4.43 (0.7-28.1)
• Suboptimal Debulking	7 (15.6)	1 (3.13)			
Differentiation/cancer grade					
• Good	6 (18.75)	7 (21.88)	0.760	1.21 (0.4-4.1)	1.09 (0.6-1.9)
• Intermediate - Poor	26 (81.25)	25 (78.12)			
Lymph nodes metastasis					
• Positive	(65.63)	11 (34.37)	0.012*	3.65 (1.3-10.2)	1.91 (1.1-3.3)
• Negative	11 (34.37)	21 (65.63)			
Ascites					
• Positive	18 (56.25)	14 (43.75)	1.000	1 (0.3-2.7)	1 (0.6-1.6)
• Negative	14 (43.75)	18 (56.25)			
Tumor size					
• ≤5 cm	6 (18.8)	8 (25)	0.545	1.44 (0.4-4.7)	1.19 (0.7-2.0)
• >5 cm	26 (81.2)	24 (75)			
Tumor residue					
• < 1cm	25 (84.4)	31 (96.87)	0.023*	8.6 (1-75.3)	4.43 (0.7-28.1)
• > 1cm	7 (15.6)	1 (3.13)			

* *p*<0.05, significant results

### AUC (area under the curve) and ROC (receiver operating characteristic) curve

**Fig. 4:**
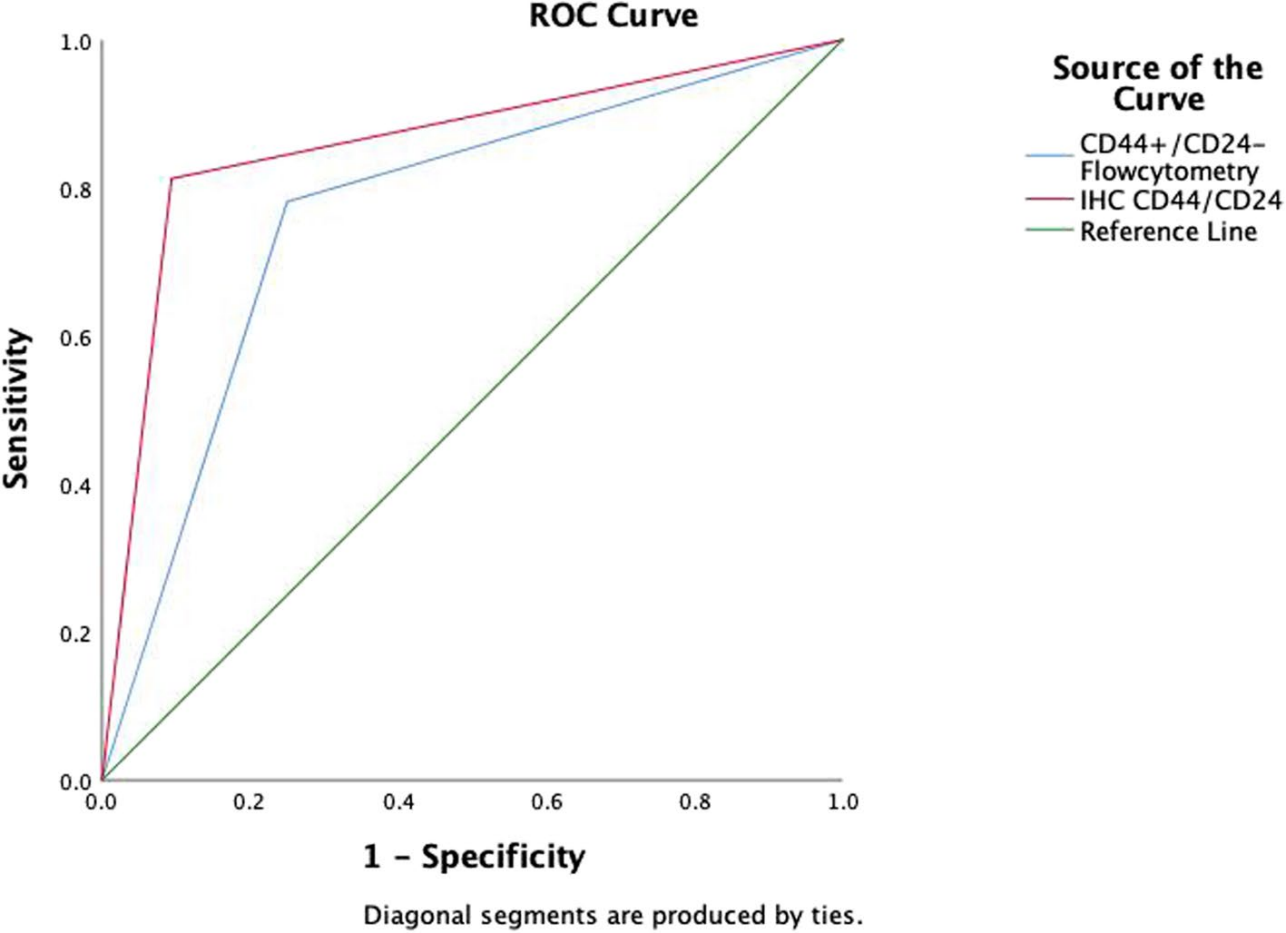
ROC curve of CSCs immunohistochemistry (red line) and flow cytometry (blue line). The red line is further to the reference means that immunohistochemistry had a better ROC curve

The immunohistochemistry of CD44^+^/CD24^−^ has a greater value, as evidenced by the AUC values (Table 3) and ROC curve (Fig. 4). The immunohistochemistry CSCs have a 96% specificity, 81% sensitivity, and a substantially excellent accuracy (AUC value 0.891, p 0.05). The sensitivity is 78%, the specificity is 75%, and the flow cytometry CSCs have a substantial fair accuracy (AUC value 0.766, p 0.05). Therefore, ovarian cancer chemoresistance may be accurately predicted using immunohistochemistry and flow cytometry CSCs.

**Table 3:** AUC comparison between immunohistochemistry and flow cytometry

Variable	AUC	SD	95% ***CI***	Sensitivity (%)	Specificity (%)	***p*** value
CD44^+^/ CD24^-^ Immunohistochemistry	0.859	0.05	0.76-0.96	81	90	0.001*
CD44^+^/ CD24^-^Flow cytometry	0.766	0.62	0.65-0.89	78	75	0.001*

*p < 0.05, significant results

### Multivariate analysis

Table 4 displays the results of the regression. A better outcome suggests that the immunohistochemistry CSCs are a better predictor.

**Table 4:** Logistic regression results comparison between immunohistochemistry and flow cytometry

No	Variables	Beta value (*β*)	Standard deviation	Wald	***p*** value	Adjusted OR	***95% CI***
**X1**	CD44^+^/ CD24^-^ immunohistochemistry	3.453	0.812	18.091	0.001*	31.590	6.435-155.078
**X1**	CD44^+^/ CD24^-^ flow cytometry	1.942	0.782	6.170	0.013	6.972	1.506-32.269
**Constant**		**-5.912 (***β*0**)**	**1**.**300**	**20.671**	**0**.000*		**-**

*p < 0.05, significant results

## Discussion

The expression of CD44^+^/CD24^-^ in ovarian cancer tissue and blood flow in ovarian cancer patients was compared for the first time in our study. Only cultured cell lines were employed in the experiments conducted in the past. Both the blood and ovarian cancer tissue have significant CSC expression. Better outcomes come from ovarian cancer tissues that exhibit CD44^+^/CD24^−^.

In both investigations, we discovered that CSCs were highly expressed in chemoresistant ovarian cancer patients. The development of ovarian cancer is linked to high CSCs expression. A strong ability for differentiation, migration, tumorigenesis, and aggressiveness was also seen in cancer stem cell tumors. The stemness characteristics of cancer cells have been described using the combination of CD44 and CD24 [11]. Ovarian cancer cells with high CD44^+^/CD24^-^ are more treatment-resistant, more capable of differentiating, and more invasive. The median PFS and cancer recurrence were both shorter in patients with greater CD44^+^/CD24^-^ ovarian cancer cells [2].

Additionally, different malignancies benefit from the prognostic value of CSCs expression. Low patient survival is associated with high CSCs expression in patients with gastric cancer [12]. High CSCs expression was also associated with breast cancer tumour development, metastasis, chemoresistance, and a poor prognosis [11, 13]. Higher levels of CD44 and CD24 expression were also seen in the pancreatic ductal adenocarcinoma and were related to its development [14].

Most cell types, including leukocytes, epithelial cells, and malignant cells have Cluster of Differentiation 44 (CD44) as cell surface adhesion receptor protein [15]. CD44 has been reported in the colorectal cancer [16], breast cancer [17], urothelial bladder cancer [18], gastric cancer [19], and pancreatic cancer [20]. According to a meta-analysis, CD44 is substantially associated with patients' survival rates being low [21]. A cell adhesion molecule in ovarian cancer is the Cluster of Differentiation 24 (CD24) [22]. Ovarian, lung, and breast cancer have all been linked to high CD24 expression [23]. Therapeutic CD24 genetic ablation may slow the growth of tumours in vivo and lengthen patient life [23].

A transmembrane protein found on cancer stem cells is called CD44. Cell proliferation, adhesion, migration, and invasion are facilitated by signalling pathways that are triggered by the binding and activation of CD44 by its ligands. These pathways also modify cytoskeletal alterations and boost cellular motility. Hyaluronic acid is the primary ligand for CD44 in cancer cells. When undergoing epithelial to mesenchymal transition (EMT) processes, epithelial ovarian cancer cells require certain stem cell characteristics. As a result, these cancer cells will express CD44 more often, be more invasive, and be more chemotherapy-resistant [24]. Inducing self-renewal processes, promoting metastasis and cell invasion are possible effects of CD44's interactions with fibroblast growth factor (FGF), osteopontin, and epidermal growth factor (EGF) [12].

Human tumours and developing B and T lymphocyte surface both express the CD24 protein. Numerous cancers, including ovarian and breast cancer, have been observed to express CD24 highly. Tumour growth, migration, and invasion are all impacted by CD24. By expelling the tumour cells from the circulation, the interaction between CD24 and P-selectin on platelets might cause metastasis. The CD24 may cause Src kinase activation and promote tumour growth. The sialic acid-binding immunoglobulin (Ig)-like lectins (Siglecs) recognize CD24 as a ligand [25]. On granulocytes, lymphocytes, and monocytes, Siglec-10 is a crucial receptor [26]. Siglec-10 and CD24's association inhibits phagocytes and encourages immunological escape mechanisms. The fundamental trait of tumour formation and recurrence is its capacity to evade the immune system [25].

No recent study has been done to specifically look at CSCs in ovarian cancer tissue and blood circulation. Further research is still required since the CD44^+^/CD24^-^ phenotype could not be the sole factor contributing to the chemoresistance, despite our significant data indicating a link between CD44+/CD24- and ovarian cancer chemoresistance. To validate these findings, external validation research is necessary.

## Conclusion

The study found a substantial correlation between ovarian cancer chemoresistance and high CSC expression. A stronger predictor of CSCs than flow cytometry is immunohistochemistry CSCs in ovarian cancer tissue.

## Data Availability

The datasets used and/or analyzed during the current study are available from the corresponding author on reasonable request.

## Abbreviations

RECIST: Response Criteria in Solid Tumors
OS: Overall Survival
PFS: Progression-free Survival
CD44: Cluster of differentiation 44
CSCs: Cancer stem cells
CD24: Cluster of differentiation 24
PBS: Phosphate-buffered Saline
EDTA: Ethylenediaminetetraacetic acid
FIGO: International Federation of Gynecology and Obstetrics
DAB: Diaminobenzene
CAT: Counterstain Kit
AUC: Area Under Curve
ROC: Receiver operating characteristic
OR: Odds Ratio
RR: Risk Ratio/relative risk
FGF: Fibroblast growth factor
EGF: Epidermal growth factor
Siglec: Sialic-acid-binding Ig-like Lectin
